# A Method to Correlate mRNA Expression Datasets Obtained from Fresh Frozen and Formalin-Fixed, Paraffin-Embedded Tissue Samples: A Matter of Thresholds

**DOI:** 10.1371/journal.pone.0144097

**Published:** 2015-12-30

**Authors:** Dana A. M. Mustafa, Anieta M. Sieuwerts, Marcel Smid, Vania de Weerd, Marcel van der Weiden, Marion E. Meijer - van Gelder, John W. M. Martens, John A. Foekens, Johan M. Kros

**Affiliations:** 1 Dept. of Pathology, Erasmus University Medical Center, Rotterdam, The Netherlands; 2 Dept. of Medical Oncology, Erasmus University Medical Center, Rotterdam, The Netherlands; University of Torino, ITALY

## Abstract

**Background:**

Gene expression profiling of tumors is a successful tool for the discovery of new cancer biomarkers and potential targets for the development of new therapeutic strategies. Reliable profiling is preferably performed on fresh frozen (FF) tissues in which the quality of nucleic acids is better preserved than in formalin-fixed paraffin-embedded (FFPE) material. However, since snap-freezing of biopsy materials is often not part of daily routine in pathology laboratories, one may have to rely on archival FFPE material. Procedures to retrieve the RNAs from FFPE materials have been developed and therefore, datasets obtained from FFPE and FF materials need to be made compatible to ensure reliable comparisons are possible.

**Aim:**

To develop an efficient method to compare gene expression profiles obtained from FFPE and FF samples using the same platform.

**Methods:**

Twenty-six FFPE-FF sample pairs of the same tumors representing various cancer types, and two FFPE-FF sample pairs of breast cancer cell lines, were included. Total RNA was extracted and gene expression profiling was carried out using Illumina’s Whole-Genome cDNA-mediated Annealing, Selection, extension and Ligation (WG-DASL) V3 arrays, enabling the simultaneous detection of 24,526 mRNA transcripts. A sample exclusion criterion was created based on the expression of 11 stably expressed reference genes. Pearson correlation at the probe level was calculated for paired FFPE-FF, and three cut-off values were chosen. Spearman correlation coefficients between the matched FFPE and FF samples were calculated for three probe lists with varying levels of significance and compared to the correlation based on all measured probes. Unsupervised hierarchical cluster analysis was performed to verify performance of the included probe lists to compare matched FPPE-FF samples.

**Results:**

Twenty-seven FFPE-FF pairs passed the sample exclusion criterion. From the profiles of 27 FFPE and FF matched samples, the best correlating probes were identified for various levels of significance (Pearson *P*<0.01, n = 1,432; *P*<0.05, n = 2,530; and *P*<0.10, n = 3,351 probes). Unsupervised hierarchical clustering of the 27 pairs using the resulting probes yielded 25, 21, and 19 correctly clustered pairs, respectively, compared to 1 pair when all probes were used.

**Conclusion:**

The proposed method enables comparison of gene expression profiles of FFPE and/or FF origin measured on the same platform.

## Background

Whole-genome microarray gene expression profiling has become an important tool for the discovery of prognostic and predictive genes for various human cancers [1,2]. Until recently, the main and preferred source of gene expression profiles was FF. In order to study larger series of tissue samples for linking expression profiles with clinical follow-up, tissue archives consisting of FFPE materials became an important source. However, the retrieval of RNA from FFPE samples is challenging [3]. RNA isolated from FFPE samples can be significantly degraded [4] and it may not contain the poly A tail for substrate binding of oligo (dT) primers to generate cDNA [5]. In addition, formalin fixation causes cross-linkage between nucleic acids and proteins [6]. Formalin reacts with nucleotides, leading to the introduction of mono-methylol groups (-CH2OH) into the bases. These alterations can interfere with enzyme activity during the process of reverse transcription, which is a critical step in microarray sample processing [7].

In breast cancer, the use of gene expression profiles to improve outcomes in defined populations was explicitly not recommended by the Evaluation of Genomic Applications in the Practice and Prevention (EGAPP) Working Group, because the evidence for the validity of the test used was considered to be inadequate [8]. In the meantime, Illumina has developed an assay called Whole-Genome cDNA-mediated Annealing, Selection, extension and Ligation (WG-DASL) specifically designed for profiling FFPE and other partially degraded RNA samples. The WG-DASL assay does not depend on poly (A)/oligo(dT) based priming in the cDNA synthesis stage. Instead, it relies on random nonamer priming. Random priming in conjugation with PCR amplification may allow for the increased detection of low abundance transcripts. In addition, the WG-DASL assay requires only short target sequences (~50 nucleotides) and therefore, degraded RNA, such as the RNA obtained from FFPE, is sufficient for this assay [9]. So far, there have been several studies addressing the applicability and suitability of the Illumina WG-DASL assay for FFPE material [9–18]. Some of these studies were performed by Illumina to demonstrate the assay reliability, reproducibility and the applicability for low RNA input [9,11]. Only a few publications focused on the direct comparison between FF and FFPE samples. Sfakianos and co-workers reported on a set of 30 matched FF-FFPE samples profiled on two different platforms (Affymetrix and Illumina’s WG-DASL) and concluded that only combinations of probes have the robustness to predict survival and classify ovarian cancer subtypes while individual probe estimates may not yield satisfactory correlations between the expression of genes isolated from FFPE and FF samples [16]. Mittempergher et al. compared 21 matched FF and FFPE gene expression profiles of breast cancer patients and found that the transcripts with higher G/C percentages of the FF and FFPE matched samples correlated best [19]. In other studies only selected gene sets were applied for the comparison of RNA expression between the FF and FFPE samples [20].

In the present study we describe a method to analyze RNA microarray data obtained from matched FFPE and FF samples of patients suffering from various types of cancers. Our method is based on data generated with Illumina’s WG-DASL BeadChips, but the principle can be applied to different gene expression platforms. Besides describing a method to define a minimum gene expression cut-off level, we developed a ruler system to reliably compare gene expression profile data obtained from FFPE and FF samples, and demonstrate its use and validity. The ruler is based on various cut-off values indicating the number of probes with a pre-specified Pearson correlation significance to be considered in the analysis.

## Results

### Sample exclusion criterion

**Fig 1:**
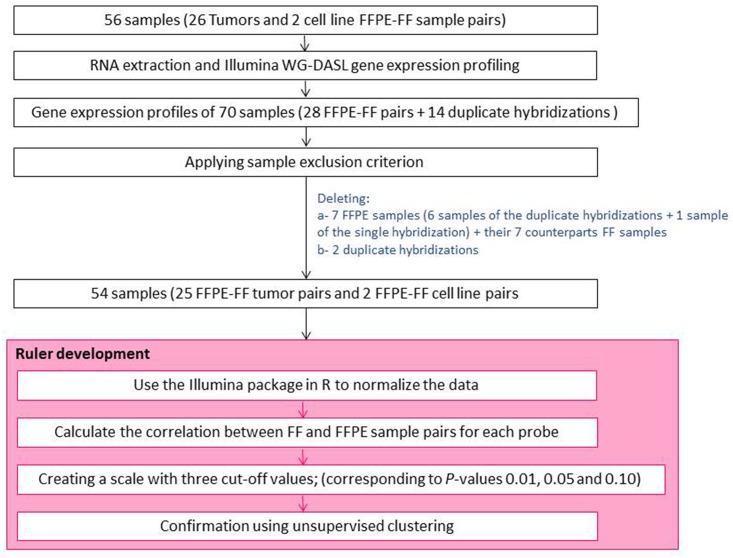
Flow chart of the method followed to analyze FFPE-FF sample pairs. 70 FFPE and FF measurements (28 FFPE-FF pairs + 14 duplicate hybridizations) were used to establish a sample-quality threshold. A threshold of 2,208 was chosen and 7 FFPE samples (6x of the duplicates and 1x of the single hybridizations) with a median below this threshold were considered of poor quality and were excluded. Because the analysis is based on FFPE-FF pairs, 7 FF pairs matching the 7 FFPE samples with low median of the reference genes were excluded from the analysis. In addition, to exclude bias in the unsupervised analysis to be performed later, the two samples that remained from the duplicate hybridizations were excluded from the analysis. The duplicate samples were selected based on their median reference gene values; duplicate samples with the lowest median reference gene values were excluded from the analysis. Due to the duplicate hybridizations which did pass the exclusion criteria, only 1 sample pair was lost to the analysis because there was no FFPE data available. The other 25 tumors and 2 cell lines had all FFPE and FF data available for further analyses In total, 54 FFPE-FF measurements (27 FFPE-FF sample pairs) were used to develop the ruler based on the reliably measured probes.

In total, 70 FFPE and FF samples (28 FFPE-FF pairs + 14 duplicate hybridizations) were available to establish a sample-quality threshold (Table 1 & Fig 1). To this end, the median fluorescence intensity of 11 reference genes was calculated (see “Materials and Methods” for the selection procedure, Table 2). A histogram of median reference gene intensity levels of all samples did not show a normal distribution (skewness -0.663) (S1A Fig). By applying an arbitrary fluorescence expression threshold of 2,208, seven FFPE samples (6 from duplicate hybridizations and 1 from single hybridization) with a median below this threshold were excluded, resulting in a close to normal distribution (skewness -0.150) (S1B Fig). Due to the duplicate hybridizations which did pass the exclusion criteria, only 1 sample pair were lost to the analysis because there was no FFPE data available. The other 25 tumors and 2 cell lines had all FFPE and FF data available for further analyses (Fig 1). In case there were duplicate hybridizations available for a sample, the one with the highest median level for the reference genes was kept. In total, 54 FFPE and FF samples (25 FFPE-FE tumor pairs and 2 FFPE-FF cell line pairs) were used for further analyses.

**Table 1:** List of FFPE-FF sample pairs included in the analysis.

Number of pairs	Tumor types	Number of hybridizations of FFPE	Number of hybridizations of FF
10	breast cancer	3 samples 2x 7 samples 1x	3 samples 2x 7 samples 1x
4	brain metastases of lung cancer	2 samples 2x 2 samples 1x	2 samples 2x 2 samples 1x
3	brain metastases of kidney cancer	1x	1x
2	brain metastases of colon cancer	1x	1x
1	brain metastases of prostate cancer	1x	1x
1	brain metastasis of esophageal cancer	1x	1x
1	brain metastasis of leiomyosarcoma	1x	1x
1	brain metastasis of ovarian cancer	1x	1x
1	brain metastasis of endometrium	1x	1x
1	Brain metastasis of melanoma	1x	1x
1	brain metastasis of ACUP	1x	1x
2	breast cancer cell lines	2 x	2 x

FFPE = formalin-fixed, paraffin-embedded; FF = fresh frozen; ACUP = adenocarcinoma of unknown primary.

**Table 2:** Stable reference gene selection based on 28 FF-FFPE pairs (56 samples).

Gene name	Illumina code	Average probe intensity	GeNorm	NormFinder		Intragroup		Intergroup	
			M-Value	SD	Accumulated SD	FF	FFPE	FF	FFPE
***ACTB*** *	ILMN_1343291	7733	0.35	0.32	0.20	0.10	0.12	-0.08	0.08
***ATP5B***	ILMN_2038778	9027	0.41	0.53	0.13	0.09	0.26	-0.32	0.32
***B2M***	ILMN_2094718	3452	0.53	0.52	0.13	0.27	0.29	-0.06	0.06
***EEF1A1*** *	ILMN_203877	19568	0.23	0.37	0.15	0.10	0.19	0.00	0.00
***GAPDH***	ILMN_1713369	9813	0.47	0.41	0.14	0.05	0.26	0.15	-0.15
***HMBS***	ILMN_1693311	5024	0.50	0.42	0.13	0.08	0.29	0.00	0.00
***HPRT1***	ILMN_2331501	3189	0.60	0.59	0.13	0.11	0.44	0.28	-0.28
***RPL13A***	ILMN_2191428	1766	0.57	0.57	0.13	0.16	0.37	0.25	-0.25
***TPT1***	ILMN_1772132	21641	0.23	0.25	0.25	0.05	0.09	-0.06	0.06
***UBB***	ILMN_1726306	6839	0.44	0.35	0.16	0.08	0.19	-0.04	0.04
***UBC***	ILMN_2148459	10474	0.29	0.33	0.17	0.05	0.15	-0.14	0.14
**median 11 selected reference genes**		**7517**	**0.29**	**0.17**	**0.17**	**-0.05**	**0.05**	**0.04**	**0.03**
***ACTN1*** *	ILMN_2216852	22697	0.84	1.10	0.14	0.54	0.63	-0.76	0.76
***AKR1D1*** *	ILMN_1722634	16970	0.80	0.88	0.13	0.09	0.57	-0.65	0.65
***ALAS1***	ILMN_1681374	3647	0.69	0.64	0.13	0.23	0.58	0.13	-0.13
***ALDOA*** *	ILMN_1801928	5516	0.98	1.54	0.16	1.80	2.24	0.61	-0.61
***G6PD***	ILMN_2051232	6690	0.92	1.39	0.15	1.02	0.46	-1.04	1.04
***GUSB***	ILMN_1669878	3186	0.73	0.79	0.13	0.08	0.84	0.41	-0.41
***HSP90AB1***	ILMN_2385647	9113	0.67	0.73	0.13	0.10	0.79	0.31	-0.31
***NUCB1*** *	ILMN_1673711	23216	0.78	0.83	0.13	0.08	0.59	-0.58	0.58
***PGK1***	ILMN_2347949	2145	0.71	0.68	0.13	0.20	0.64	-0.23	0.23
***PUM1***	ILMN_2056975	5360	0.75	0.83	0.13	0.09	0.56	0.58	-0.58
***SDHA***	ILMN_1783424	3830	0.87	1.15	0.14	0.19	1.68	0.61	-0.61
***TMBIM6***	ILMN_2157277	11760	0.65	0.64	0.13	0.16	0.59	0.22	-0.22
***YWHAZ***	ILMN_2232177	9990	0.62	0.62	0.13	0.06	0.38	0.40	-0.40

All samples from Table 1 were used. In case of duplicate hybridizations, the sample with the highest median value for the reference genes was selected. GeNorm and NormFinder software were used to calculate the stability of genes. M-value; the average expression stability value, for which an arbitrarily cut-off at 0.6 was used to assign genes as stably expressed or not. *ACTB* = Actin-Beta; *ATP5B* = ATP Synthase Subunit Beta; B2M = beta-2-microglobin; *EEF1A1* = eukaryotic translation elongation factor 1 alpha 1; *GAPDH* = Glyceraldehyde-3-Phosphate Dehydrogenase; *HMBS* = hydroxymethylbilane synthase; *HPRT1* = hypoxanthine phosphoribosyltransferase 1; *RPL13A* = ribosomal protein L13a; *TPT1* = tumor protein, translationally-controlled 1; *UBB* = ubiquitin B; *UBC* = ubiquitin C; *ACTN1* = actinin, alpha 1; *AKR1D1* = aldo-keto reductase family 1, member D1; *ALAS1* = aminolevulinate, delta-, synthase 1; *ALDOA* = aldolase A, fructose-bisphosphate; *G6PD* = glucose-6-phosphate dehydrogenase; *GUSB* = glucuronidase, beta; *HSP90AB1* = heat shock protein 90kDa alpha (cytosolic), class B member 1; *NUCB1* = nucleobindin 1; *PGK1* = phosphoglycerate kinase 1; *PUM1* = pumilio RNA-binding family member 1; *SDHA* = succinate dehydrogenase complex, subunit A, flavoprotein (Fp); *TMBIM6* = transmembrane BAX inhibitor motif containing 6; *YWHAZ* = tyrosine 3-monooxygenase/tryptophan 5-monooxygenase activation protein, zeta.

* Belong to the housekeeping gene set of Illumina.

### Selection of probes

For each individual probe the expression levels measured in 27 FFPE samples were correlated with the case-matched FF levels. Three Pearson R cut-off *P*-values (*P*<0.01, *P*<0.05 and *P*<0.10) were used for the inclusion of probes from the total set of 24,526 probes. The significance levels of *P*<0.01, *P*<0.05 and *P*<0.10 allowed the inclusion of 1,697, 3,016 and 3,982 probes for analysis, respectively.

Next, the slope and y-axis intercept were calculated for each probe included at the individual 3 cut-offs. The mean of all slopes and the mean of all y-axis intercepts were calculated. All outlier probes (probes with slopes and/or y-intercept values outside of the mean ± one standard deviation) were deleted. This yielded 1,432 out of 1,697 probes at *P*<0.01; 2,530 out of 3,016 probes at *P*<0.05; and 3,351 out of 3,982 probes at *P*<0.10. These 3 lists consisted of probes with minimal variation in expression levels between FFPE and FF materials.

### Confirmation: comparing FFPE-FF sample pairs

Using the 3 probe lists, the Spearman rank correlation (Rs) was calculated between the expression of the probes of 27 sample pairs. The Rs ranged from Rs 0.74 to 0.99 using the 1,432 probes of the *P*<0.01 list; from 0.66 to 0.99 using 2,530 probes of the *P*<0.05 list; and from 0.63 to 0.99 using 3,351 probes *of the P*<0.10 list. The Rs between the expression of all probes (24,526) for the 27 FF and FFPE samples ranged from 0.55 to 0.93 (Table 3).

**Table 3:** Correlation between FFPE-FF sample pairs.

		Number of Probes			
	Sample pairs	1,432 *P*<0.01	2,530 *P*<0.05	3,351*P*<0.10	24,526 *P* N.A
1	Breast Ca. 1	0.78	0.70	0.66	0.57
2	Breast Ca. 2	0.88	0.84	0.83	0.81
3	Breast Ca. 3	0.94	0.93	0.92	0.90
4	Breast Ca. 4	0.87	0.83	0.81	0.76
5	Breast Ca. 5	0.92	0.88	0.87	0.82
6	Breast Ca. 6	0.86	0.82	0.81	0.76
7	Breast Ca. 7	0.90	0.86	0.85	0.81
8	Breast Ca. 8	0.81	0.80	0.80	0.90
9	Breast Ca. 9	0.99	0.99	0.99	0.99
10	Breast Ca. 10	0.84	0.80	0.78	0.79
11	Brain meta. lung Ca. 1	0.86	0.83	0.81	0.76
12	Brain meta. Lung Ca. 2	0.85	0.80	0.78	0.75
13	Brain meta. Lung Ca. 3	0.76	0.72	0.72	0.74
14	Brain meta. Lung Ca. 4	0.86	0.84	0.83	0.81
15	Brain meta. colon Ca. 1	0.90	0.87	0.86	0.82
16	Brain meta. colon Ca. 2	0.79	0.73	0.70	0.55
17	Brain meta. kidney Ca. 1	0.88	0.84	0.83	0.74
18	Brain meta. kidney Ca. 2	0.83	0.78	0.77	0.67
19	Brain meta. kidney Ca. 3	0.90	0.86	0.85	0.77
20	Brain meta. prostate Ca.	0.87	0.85	0.83	0.73
21	Brain meta. oesophagus Ca.	0.84	0.79	0.78	0.69
22	Brain meta. Leiomyosarcoma	0.74	0.66	0.63	0.55
23	Brain meta. ovarian Ca.	0.83	0.80	0.78	0.75
24	Brain meta. endometrium Ca.	0.87	0.83	0.81	0.76
25	Brain meta. ACUP	0.83	0.79	0.78	0.75
26	Breast cell-line [MDA-MB-231]	0.96	0.96	0.96	0.93
27	Breast cell-line [SKBR3]	0.95	0.94	0.94	0.90
**Number of matched pairs following unsupervised hierarchical clustering**		25/27	21/27	19/27	1/27

Rows 1 through 27 represent the paired samples of which the FFPE and FF parts were separately run on the Illumina WG-DASL V3 platform. The right four columns show the Spearman rank correlation coefficients between the expression values of the FF and FFPE materials for the distinct *P*-values (0.01; 0.05; 0.10 and N.A. = Not Applicable). The upper row shows the numbers of probes included for the distinct levels of significance. The number of matched pairs following unsupervised hierarchical clustering is shown in the bottom row.

**Fig 2:**
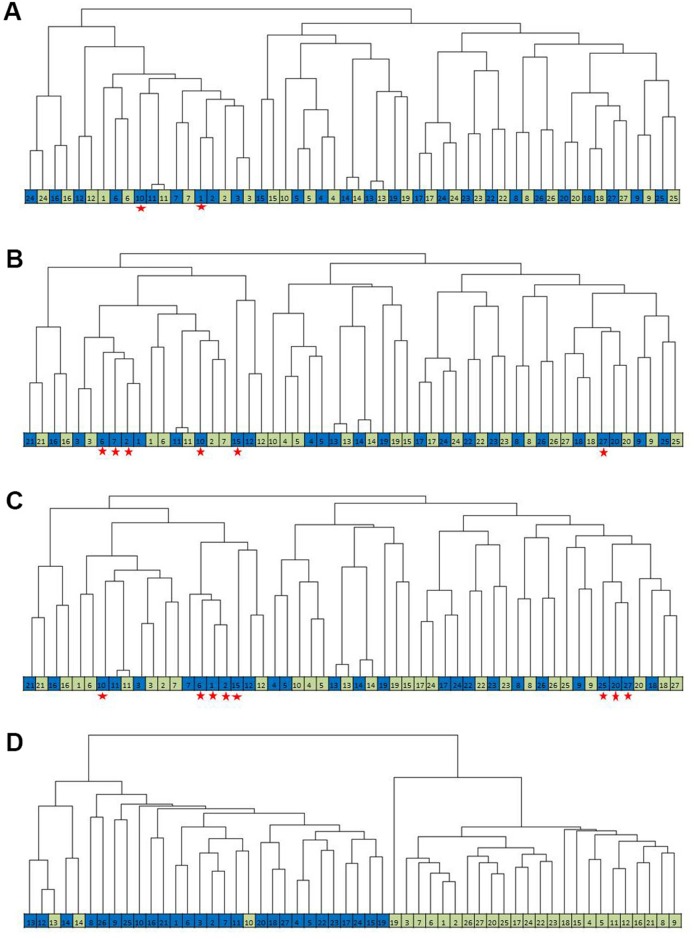
Dendrograms of matched FF and FFPE pairs at the three cut-off values. **(A)** Clusters based on 1,432 probes (*P*<0.01): only 2 FFPE samples did not cluster with their FF counter pairs. (**B)** Clusters based on 2,530 probes (P<0.05): 6 FFPE samples did not cluster with their FF counter pairs. **(C)** Clusters based on 3,351 probes (P<0.1): 8 FFPE samples did not cluster with their FF counter pair sample. **(D)** Clusters based on all the 24,526 profiled probes: the main two arms of the dendrogram are based on the FFPE or FF samples. Each square represents a sample; Blue: FFPE; Green: FF, Red asterix indicates the FFPE samples that did not cluster with their FF counterpart.

Next, the 27 pairs were used for a clustering analysis. Unsupervised hierarchical clustering using 1,432 probes (*P*<0.01) showed that 25 out of the 27 pairs clustered correctly together (Table 3 **bottom row and** Fig 2A). The number of matched pairs decreased when using the additional probes from different cut-off values: 22/27 pairs using 2,530 probes (*P*<0.05) (Fig 2B), and 19/27 pairs using 3,351 probes (*P*<0.10) (Fig 2C). In contrast, unsupervised clustering of the 24,652 probes revealed a dominant effect of tissue handling: almost all FF samples clustered separately from the FFPE samples while only one paired FFPE-FF sample clustered together (Fig 2D).

### Tissue specificity

To investigate a possible influence caused by the origin of the tissues on the gene expression correlation between FFPE and FF samples, an identical analysis was performed restricted to the FFPE-FF sample pairs of a single tumor type, namely 10 primary breast cancers and two breast cancer cell lines. The same probe selection procedure as described above was followed, resulting in 1,118 reliable probes at *P*<0.01, 2,285 probes at *P*<0.05, and 3,937 probes at *P*<0.10. The Spearman rank correlation coefficients and the clustering improved slightly for some cases, but overall the results matched those of the entire set of 27 sample pairs (S2 Table).

### Effect of normalization

The effect of data-normalization on the correlation was investigated by normalizing the data obtained with the 12 FFPE and the 12 FF breast cancer samples separately in Lumi R, prior to the correlation analyses, instead of normalizing all 24 samples combined. The correlation between the expression patterns of the FFPE and FF samples was calculated starting from the probe selection procedure. The individual normalization resulted in a slightly higher number of reliable probes at the different cut-offs, but did not influence the outcome of the Spearman rank correlation analysis between the FFPE and FF sample pairs (S3 Table).

### Reproducibility of the method in FFPE samples only

To study the reproducibility of the analysis of the FFPE samples only, 15 FFPE samples of various tumor types and 2 breast cancer cell lines were measured twice (total of 34 hybridizations, S1 Table). Following the exclusion criterion based on the minimum median expression of 11 reference genes, 14 duplicate samples (28 hybridizations) were included in the analysis (S2 Fig). Data were analyzed following the same method as described above. The Spearman rank correlation coefficients ranged from 0.55 to 0.99 when all 24,526 probes were taken into account. The Rs ranged from 0.88 to 0.99 at *P*<0.01 (2,732 probes), 0.84 to 0.99 at *P*<0.05 (4,700 probes), and 0.83 to 0.99 at *P*<0.10 (5,833 probes) (S4 Table). Hierarchical clustering showed that all FFPE samples clustered with their duplicate when using the 3 cut-off values, but also when using all 24,526 probes (S4 Table, **bottom row**).

## Discussion

Because most of the available data on RNA expression profiling in the literature has been generated from fresh frozen samples, it is important to establish methods for reliably comparing gene expression profiles derived from FFPE and FF samples. In this study we investigated the effects of tissue handling prior to processing for the RNA expression array technology and calculated the correlation coefficients between the gene expression profiles at various levels of stringency. Gene expression profiles obtained from matched FFPE and FF tumor and normal adjacent lung tissue samples showed that the number of reliably measured probes was slightly higher in FF samples as compared to FFPE samples [9]. The number of reliably measured probes (*P*<0.01 calculated by GenomeStudio for the approximately 30 beads tagged with these probes) in our study was also higher in the FF samples compared with the FFPE samples for 24/27 pairs. Only three FFPE samples had a higher number of reliably measured probes compared with the FF samples.

The median expression of 11 reference genes was used to estimate the quality and quantity of the input RNA. We defined a threshold for including samples based on the normal distribution of the median expression levels of these 11 reference genes. In earlier attempts of Thomas et al., to match FF and FFPE gene expression data, the use of “percentage of genes present” was recommended [2]. In that study however, only a single reference gene (*DDX5*) was used to judge the quality of the expression profiles and a threshold based on the expression levels of this gene for discarding samples of poor quality was recommended [2]. Some studies did not specify a method to check the quality of the expression profiles [19–21], or only probes with *P*-values of less than 0.05 were included [22]. Another study, which used Affymetrix gene expression profiles of FFPE samples, showed that almost half of the profiled samples (19/34) failed to meet the stringent quality control criteria described by Affymetrix [23]. In our view, using the median expression of multiple reference genes that are stably expressed in both FF and FFPE samples, rather than only one gene, provides a better basis for judging the quality of profiled gene expression arrays, especially for mixed FFPE and FF samples.

The present analysis was performed at the level of the probes, not genes, as some probes may be better equipped to quantify the levels of a certain gene over different cohorts than others. Using the average value of all probes that hybridize to a specific gene transcript may thus affect the validity of the measurement. Our results indicate that simply including all measured probes when analyzing data obtained from RNA expression profiling of FFPE and FF samples may lead to false discoveries, which will be impossible to validate. The unsupervised hierarchical clustering using all measured probes showed a matching of samples that was almost solely based on methods of tissue preparation (FFPE or FF) rather than on biological similarities (Fig 2D). Obviously, sensible comparisons of expression data from various sources, including FFPE and FF procedures, can only be made if appropriate probes which correlate well between specimens from different origin are considered. Increased stringency of inclusion of probes correlating well between matched FFPE and FF specimens will inevitably lead to a significant reduction of probes to be interpreted. We eliminated outlier probes based on the slope and the y- axis intercept of the Pearson correlation plot between FFPE and FF paired samples, after which at the level of *P*<0.01 only 1,432 (5.8%) of all probes passed the threshold of reliability. This number increased to more than 10.3% (2,530 probes) when the *P*-value was raised to 0.05 and increased to 13.7% (3,351 probes) when the *P*-value was further raised to 0.10. At *P*<0.01 the correlation between the pairs ranged between 0.74–0.99 and the cluster analysis showed a match of 25 out of the 27 pairs. The 2 non-matching samples in this analysis clustered to another breast cancer sample, which may be explained by the heterogeneity within the tissue [10,19,24]. The number of paired samples clustering together decreased after increasing the number of probes included in the analysis. Clearly, the loss of information due to reduction of number of probes to be taken into account is compensated by the gain in reliability of comparing FFPE with FF samples. This way, stringent cut-off values (for example *P*<0.01) can reduce the number of validation experiments. On the other hand, the more stringent *P*-values leading to relative low numbers of probes to be taken into account will lead to a reduction in sensitivity of discovery. Therefore, we recommend that the choice of the cut-off *P*-value for selecting reliably measured probes depends on the resources and the number of samples used for both data analysis and validation studies. If the validation group is relatively large, a researcher may choose to use *p* < 0.1. However, we recommend using the more stringent cut-off (*P*<0.01) if the validation experiments and the sample size are limited. Probes that were not included in the gene lists are too different between an FF or FFPE measurement of the same sample. Such genes are vulnerable to either the FF or FFPE pretreatments, and other methods than microarray analysis to obtain expression level data of such genes are also at risk of measuring different levels in the FF or FFPE sample.

Several methods of analyzing Illumina WG-DASL gene expression profiles of matched FFPE and FF samples have been proposed previously. Direct comparisons between the expression data from FFPE and FF samples of neoplastic and normal lung samples yielded correlations around 0.7 [9]. Our approach yields a considerable improvement of correlation between FFPE and FF material, with an average of 0.86 for the Pearson correlation when using the most strict cut-off value (*P*<0.01) (Table 3) and the average correlation coefficients are still 0.83 and 0.81 at the cut-off values of *P*<0.05 and *P*<0.10, respectively, as compared to 0.77 when all probes are included. The improvement of the correlation is not influenced by the quality of the probes because the results of repetitive measuring of probes of insufficient quality are inconsistent and would negatively influence the correlation coefficients. In another study in which the expression results of FFPE and matched FF samples of 20 breast cancers were compared, probes with high expression variance were included. This method resulted in using 5,444 (20%) of the total amount of probes [19]. This approach is comparable to our method, allowing the inclusion of 3,351 probes (13.7%) at *P*<0.10. However, only 19/27 pairs clustered together when applying the unsupervised hierarchical clustering. Therefore, despite the increase of probes to be taken into account, more extensive validation studies are necessary to confirm the findings.

The effects of the age of the paraffin blocks on RNA quality has been addressed previously and in general, RNA degradation is more extensive in older paraffin blocks [25,26]. In our series some samples were over 20 years old. In addition, samples were collected from various centers in The Netherlands and therefore, fixation was not carried out in a uniform fashion. By using the quality control as described, the effects of age and quality can be effectively compensated for. Separate normalization of FFPE samples and FF samples was recommended previously for profiling samples on the Affymetrix platform [27]. It was shown that normalizing the gene expression profiles of FFPE and FF samples together can skew the normalized data. Following the data analysis described in this study, separate normalization of the FFPE and FF samples will not measurably affect or skew the data. Repeating our analysis after separate normalization of the FFPE and FF samples, correlation coefficients slightly improved (S3 Table), but the number of matched pairs identified by unsupervised hierarchical clustering was not affected by the separate normalization.

The accuracy and sensitivity of profiling FFPE samples by repetitive measuring of samples using various platforms like Illumina or Affymetrix has been scrutinized before [2,9,13,19,20,22,28,29]. In this study we profiled 17 FFPE samples in duplicate. The samples were subjected to the exclusion criterion and the expression data were correlated, yielding average Spearman rank correlations of 0.96 ± 0.03 when using the probes at *P*-value <0.01 as compared to 0.90 ± 0.11 when including all probes (S4 Table). Similar improvements were obtained when we used the FF samples for the duplicate studies (data not shown). All values obtained from duplicate measurements resulted in matched clustering using the three cut-off values. These results again highlight the accuracy and sensitivity of the current method to analyze Illumina WG-DASL platform data. Our analyses indicate that selecting the most correlated probes to analyze profiled data obtained from FFPE samples only is a reliable method. However, this selection of the most correlated probes may not be crucial to obtain good data analysis results that can be validated.

## Conclusion

**Fig 3:**
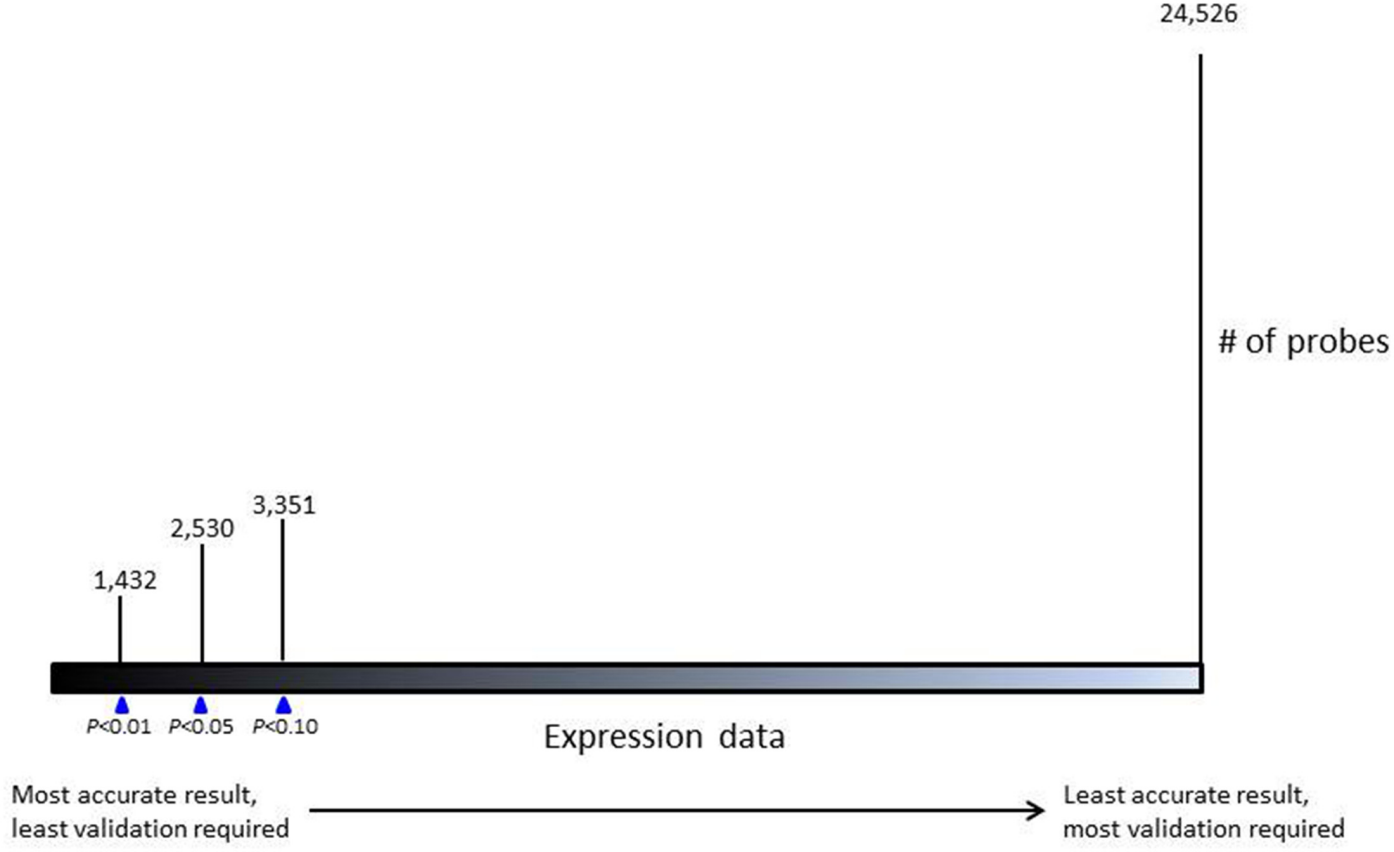
Ruler of the three cut-off values used to compare the gene expression profiles of FFPE and FF samples. Each Pearson *P*-value results in different numbers of probes to be included for further analysis.

In summary, we describe a novel and simple method to reliably compare gene expression data derived from FFPE and FF samples, using probes with various levels of correlation strength (*P*-value <0.01, <0.05 and <0.10). The reliable probes identified by this method will vary between different datasets. The method is applicable to various platforms, and is successful in selecting probes yielding consistent signals across tumor types, and proved independent of the normalization pre-processing. The number of probes to include and the comparison between FFPE and FF samples is dependent on individual criteria for significance. Applying this method will enable researchers to profile the archived FFPE samples and compare the data to already available profiled data obtained from FF samples. Our data however also show that, in order to analyze expression data from FFPE and FF samples, a trade-off (Fig 3) between the number of included probes and the ability to accurately compare FFPE and FF samples will apply.

## Materials and Methods

### Tissue sample selection

Paired FFPE and FF tissue specimens of 26 tumors of various kinds and two cultured breast cancer cell lines (MDA-MB-231 and SKBR3) were selected. Of 5 patients and 2 cell lines enough RNA material was available from both FFPE and FE samples to perform duplicate hybridizations using Illumina WG-DASL (Illumina, Scoresby, VIC, Australia). In total, 70 measurements were carried out [26*2 (FFPE-FF tumor sample pairs) + 2*2 (FFPE-FF cell line pairs) + 7*2 (duplicate hybridizations: 3 FFPE-FF breast cancer pairs, 2 FFPE-FF brain metastases of lung pairs, and 2 FFPE-FF cell line pairs)] (Table 1). In addition, to study the reproducibility of the established analysis workflow, following independent hybridizations RNA of 15 FFPE samples and 2 FFPE breast cancer cell lines (MDA-MB-231 and SKBR3) was profiled twice (S1 Table). This study was approved by the Medical Ethics Committee of the Erasmus Medical Center, Rotterdam, The Netherlands (MEC 02·953) and performed in adherence to the Code of Conduct of the Federation of Medical Scientific Societies in the Netherlands (http://www.fmwv.nl/). Permission to use the biomaterials for this investigation was obtained in writing. The institutional review board or ethics committee waived the need for consent (according to the bylaw "code goes gebruik biologisch restmateriaal).

### Morphological assessment

Five μm H&E sections from each sample were prepared before and after sectioning for RNA isolation. To ascertain similar morphology of the sections used for the FFPE and matched FF RNA isolation, the origins of the primary tumor, the percentages of the invasive tumor cells, inflammatory infiltrates and the presence of necrosis were taken into consideration (JMK). Only RNA isolated from sections with similar morphology was included for the matched FFPE-FF comparisons.

### RNA extraction and purification

Total RNA from FF tissue samples was, depending on the size of the sample, extracted from 20–30 sections of 30 μm using RNABee reagent according the supplier's instructions (Campro Scientific, Veenendaal, The Netherlands). Depending on the size of the FFPE samples, RNA was extracted from 3 to 6 x 20 μm sections. Following paraffin removal with xylene the high-pure RNA paraffin kit was used according the supplier's instructions (Roche, Mannheim, Germany). Following isolation, RNA was stored in RNase/DNase-free water at -80°C.

### RNA quality control

The quantity and quality of the isolated RNA was monitored by UV spectroscopy and by examination of rRNA bands after agarose gel electrophoresis. Samples were excluded if the yield did not reach the minimum requirement of 500 ng available for the FF samples and 1,000 ng for the FFPE samples. As an inner-assay control, 500 ng of FF RNA isolated from a pool of several cultured breast cancer cell lines was used. The inner-assay control was used to monitor the assay performance and to evaluate the inter-assay BeadChip variability for each experiment.

### Gene expression profiles

Illumina WG-DASL assay is an array-based method for expression profiling of partially degraded RNA molecules such as those isolated from FFPE samples. In the assay 24,526 annotated transcripts corresponding to 18,391 unique genes are measured. The WG-DASL assay was performed according to the manufacturer’s instructions. In brief, 500 ng total RNA was used from FF samples and 1,000 ng of total RNA from FFPE samples. 500 ng of total RNA from the inner-assay control was included in each measurement. Total RNA was converted to cDNA using biotinylated oligo-dT_18_ and random nonamer primers. The biotinylated cDNA was annealed to the DASL Assay Pool (DAP) probe groups, which contain oligonucleotides specifically designed to interrogate each target sequence of the transcript. The DAP was annealed to targeted cDNA during a 16 hours temperature gradient (70° to 37°C) incubation. Hybridization of these oligonucleotides to the targeted cDNA site, followed by enzymatic extension and ligation was used to create a PCR template that was amplified with a set of universal PCR primers [30]. Cy3-coupled primers were used to facilitate the precipitation of the single stranded labeled products, which were hybridized to the whole genome Human Ref8_V3_BeadChips containing 8 identical microarrays each. The microarrays were scanned using a confocal type imaging system with Cy3 (532 nm) laser illumination (Illumina I-scan reader N0262). Fluorescent intensities were read and images were extracted using software version 1.8.13.5. Each sequence type is represented by an average of 30 beads on the array.

### Data Analysis

Scanned data were uploaded into GenomeStudio software version 2011.1 via the Whole Genome DASL gene expression module for further analysis. The average signal, detection *P*-value, bead standard error and average beads were used to quantile normalize the data in the statistical language R (www.r-project.org/) using the “Lumi” package [31].

In order to reach a reliable method to analyze profiled data from FFPE and FF samples, three major analyses were performed: sample exclusion criteria, probe selection and confirmation.

### Sample exclusion criterion

In addition to the 6 Housekeeping probe sets selected by Illumina, 18 reference genes were selected based on the literature and tested for their signal intensity and stable expression level between both FFPE and FF samples on this platform, using GeNorm [32] and NormFinder [33] software packages. Eleven out of the 24 genes showed a stable expression in both FFPE and FF samples with a minimum average expression stability value of 0.6 (Table 2). The median expression level of the 11 reference genes was calculated and used to establish a minimum lower threshold. Samples that did not meet the threshold for these 11 reference genes were excluded (see Results section).

### Probe selection

Expression levels, normalized by the ‘Lumi’ package, were used to calculate the correlation coefficient (Pearson R2 correlation) at the individual probe level for all paired FFPE and FF samples (n = 54; 27 pairs) that were left after applying the sample exclusion criterion. Various levels of significance were used (*P*<0.01 [R2>0.49]; *P*<0.05 [R2>0.38] and *P*<0.10 [R2>0.32], respectively). To calculate the accuracy of the associations between the individual probe values of FFPE and FF samples, the slope and the y-intercept were calculated for each probe at the different cut-offs. The mean of all slopes and the mean of all y-intercepts were calculated and all outlier probes (probes with expression values not within the mean ± standard deviation) were disregarded.

### Confirmation

Unsupervised clustering was performed using Cluster 3.0 using the correlation (uncentered) average linkage method and Java Treeview 1.1.6 R2 was used for visualization [34].

## Supporting Information

S1 Fig Histogram of the log-transformed median expression of 11 reference genes prior to- and following- the omission of samples with relatively low expression levels. **(A)** Implementing all 70 samples resulted in a distribution with a skewness of -0.663. **(B)** Following the deletion of 7 samples with a median < 2,208 (natural log-transformed (LN = 7.7)) fluorescent expression level for the 11 reference genes the skew was reduced to an acceptable level (skewness of -0.150). The red line represents a normal distribution of the values.
(TIF)

S2 Fig Flow chart of the method followed for the duplicate expression measurements of FFPE samples. 34 FFPE measurements (17 duplicate FFPE samples) were used to apply the sample exclusion criteria that was established earlier in the study based on a threshold of 2,208. Three FFPE samples with a median reference gene signal below this threshold were of poor quality and were excluded. Because the analysis is based on FFPE duplicate measurements, the duplicate measures of 3 FFPE samples of poor quality were excluded from the analysis. In total, 28 FFPE measurements (14 duplicate FFPE samples) were used to identify the reliably measured probes.
(TIF)

S1 Table List of FFPE samples profiled in duplicate. FFPE = formalin-fixed, paraffin-embedded; ACUP = adenocarcinoma of unknown primary.
(DOCX)

S2 Table Effect of tissue origin on the Spearman rank correlation between FF and FFPE samples. Rows 1 through 12 represent breast cancer paired samples of which the FFPE and FF parts were separately run on the Illumina WG-DASL V3 platform. The right four columns show the Spearman rank correlation coefficient between the expression values of the FF and FFPE materials for various *P*-values (0.01; 0.05; 0.10 and N.A. = Not Applicable). The upper row shows the numbers of probes included for the distinct levels of significance. The number of matched pairs following unsupervised hierarchical clustering is shown in the bottom row.
(DOCX)

S3 Table Effect of normalization on the Spearman rank correlation between FF and FFPE samples. Rows 1 through 12 represent breast cancer FFPE and FF sample pairs which were separately run on the Illumina WG-DASL V3 platform. The normalization was performed separately for the FF and FFPE samples. The right four columns show the correlation coefficient (Spearman correlation) between the expression values of FF and FFPE materials for various p-values (0.01; 0.05; 0.10 and N.A. = Not Applicable). The upper row shows the numbers of probes included for the distinct levels of significance. The number of matched pairs following unsupervised hierarchical clustering is shown in the bottom row.
(DOCX)

S4 Table Reproducibility among repeated measurements of FFPE samples. Rows 1 through 14 represent the FFPE samples that were profiled twice on the Illumina WG-DASL V3 platform. The right four columns show the correlation coefficient (Spearman correlation) between the expression data of the duplicates for various p-values (0.01; 0.05; 0.10 and N.A. = Not Applicable). The upper row shows the numbers of probes included for the distinct levels of significance. The number of matched pairs following unsupervised hierarchical clustering is shown in the bottom row.
(DOCX)

## Abbreviations

FFPE: Formalin-Fixed, Paraffin-Embedded
FF: Fresh Frozen
WG-DASL: Whole-Genome cDNA-mediated Annealing, Selection, extension and Ligation
