# Supplementary figures and images for: A Method to Correlate mRNA Expression Datasets Obtained from Fresh Frozen and Formalin-Fixed, Paraffin-Embedded Tissue Samples: A Matter of Thresholds
Source: PLoS One. 2015 Dec 30;10(12):e0144097. doi: 10.1371/journal.pone.0144097 (PMC4696787; doi:10.1371/journal.pone.0144097)

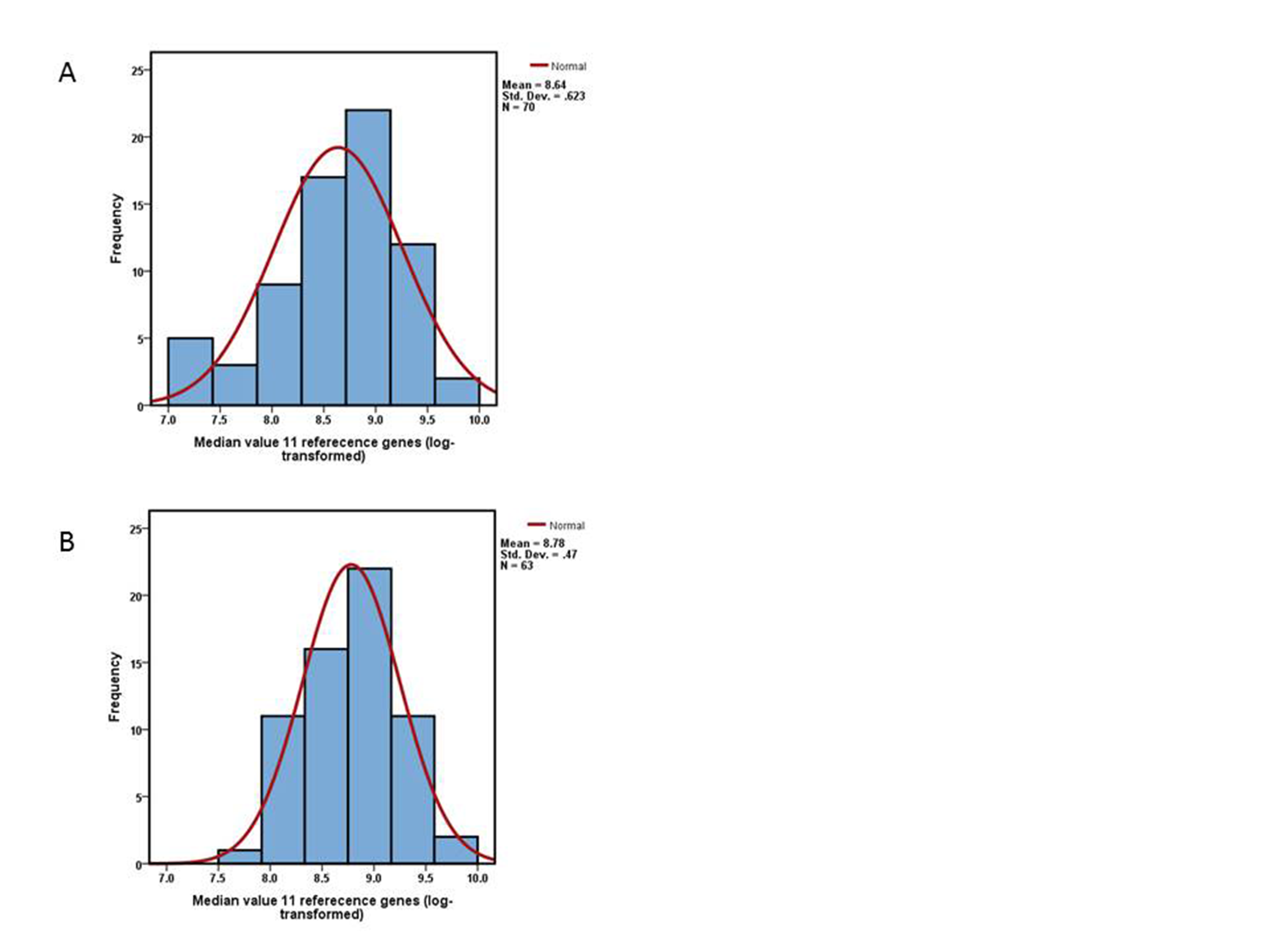

Supplement: S1 Fig — (A) Implementing all 70 samples resulted in a distribution with a skewness of -0.663. (B) Following the deletion of 7 samples with a median < 2,208 (natural log-transformed (LN = 7.7)) fluorescent expression level for the 11 reference genes the skew was reduced to an acceptable level (skewness of -0.150). The red line represents a normal distribution of the values. (TIF) [file pone.0144097.s001.tif]

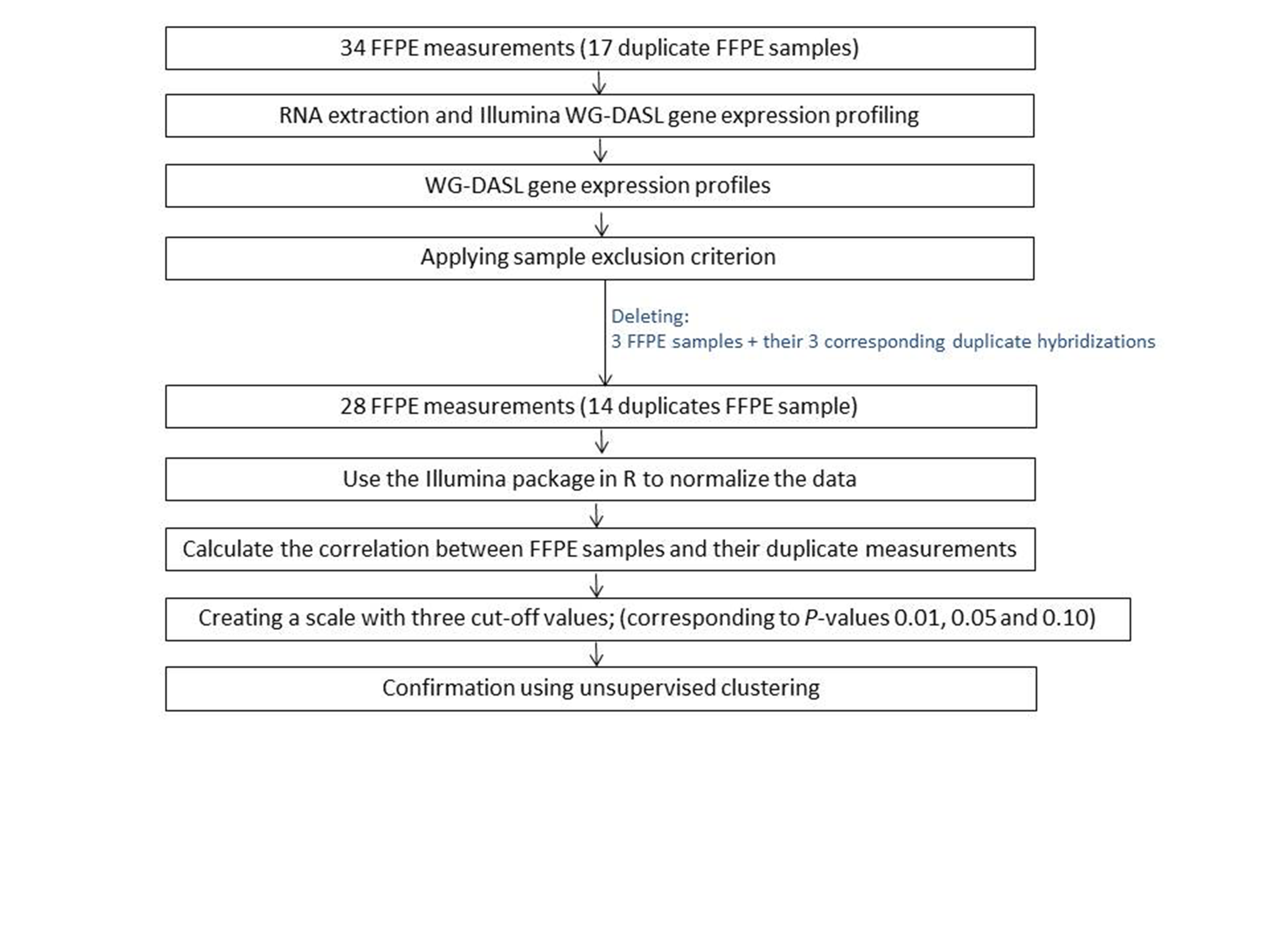

Supplement: S2 Fig — 34 FFPE measurements (17 duplicate FFPE samples) were used to apply the sample exclusion criteria that was established earlier in the study based on a threshold of 2,208. Three FFPE samples with a median reference gene signal below this threshold were of poor quality and were excluded. Because the analysis is based on FFPE duplicate measurements, the duplicate measures of 3 FFPE samples of poor quality were excluded from the analysis. In total, 28 FFPE measurements (14 duplicate FFPE samples) were used to identify the reliably measured probes. (TIF) [file pone.0144097.s002.tif]
