# Supplementary material for: A Method to Correlate mRNA Expression Datasets Obtained from Fresh Frozen and Formalin-Fixed, Paraffin-Embedded Tissue Samples: A Matter of Thresholds
Source: PLoS One. 2015 Dec 30;10(12):e0144097. doi: 10.1371/journal.pone.0144097 (PMC4696787; doi:10.1371/journal.pone.0144097)
Supplement: S1 Table — FFPE = formalin-fixed, paraffin-embedded; ACUP = adenocarcinoma of unknown primary. (DOCX) [file pone.0144097.s003.docx]

S1 Table

Title: List of FFPE samples profiled in duplicate

| **Numbers** | **Tumor types** | **Number of hybridizations of FFPE** |
| --- | --- | --- |
| 7 | brain metastasis of lung cancer | 2x |
| 2 | brain metastasis of ACUP | 2x |
| 1 | brain metastasis of breast cancer | 2x |
| 1 | brain metastasis of esophageal cancer | 2x |
| 1 | brain metastasis of kidney cancer | 2x |
| 1 | brain metastasis of prostate cancer | 2x |
| 1 | brain metastasis of endometrium | 2x |
| 1 | brain metastasis leiomyosarcoma | 2x |
| 2 | breast cancer cell lines | 2x |

Legend S1 Table:

FFPE = formalin-fixed, paraffin-embedded; ACUP = adenocarcinoma of unknown primary
