# Supplementary material for: A Method to Correlate mRNA Expression Datasets Obtained from Fresh Frozen and Formalin-Fixed, Paraffin-Embedded Tissue Samples: A Matter of Thresholds
Source: PLoS One. 2015 Dec 30;10(12):e0144097. doi: 10.1371/journal.pone.0144097 (PMC4696787; doi:10.1371/journal.pone.0144097)
Supplement: S2 Table — Rows 1 through 12 represent breast cancer paired samples of which the FFPE and FF parts were separately run on the Illumina WG-DASL V3 platform. The right four columns show the Spearman rank correlation coefficient between the expression values of the FF and FFPE materials for various P-values (0.01; 0.05; 0.10 and N.A. = Not Applicable). The upper row shows the numbers of probes included for the distinct levels of significance. The number of matched pairs following unsupervised hierarchical clustering is shown in the bottom row. (DOCX) [file pone.0144097.s004.docx]

S2 Table

Title: Effect of tissue origin on the Spearman rank correlation between FF and FFPE samples

|  | **Number of Probes** | | | |
| --- | --- | --- | --- | --- |
| **Sample pairs** | **1,118**  ***P*<0.01** | **2,285**  ***P*<0.05** | **3,937**  ***P*<0.10** | **24,526**  ***P* N.A.** |
| Breast Ca. 1 | 0.92 | 0.86 | 0.83 | 0.57 |
| Breast Ca. 2 | 0.95 | 0.92 | 0.91 | 0.82 |
| Breast Ca. 3 | 0.97 | 0.96 | 0.95 | 0.90 |
| Breast Ca. 4 | 0.94 | 0.91 | 0.88 | 0.76 |
| Breast Ca. 5 | 0.95 | 0.93 | 0.91 | 0.82 |
| Breast Ca. 6 | 0.94 | 0.91 | 0.89 | 0.77 |
| Breast Ca. 7 | 0.95 | 0.93 | 0.91 | 0.81 |
| Breast Ca. 8 | 0.93 | 0.91 | 0.90 | 0.90 |
| Breast Ca. 9 | 0.99 | 0.99 | 0.99 | 0.99 |
| Breast Ca. 10 | 0.93 | 0.90 | 0.86 | 0.78 |
| Breast cell-line [MDA-MB-231] | 0.97 | 0.97 | 0.96 | 0.93 |
| Breast cell-line [SKBR3] | 0.96 | 0.95 | 0.94 | 0.91 |

| **Number of clustered pairs**  **following unsupervised hierarchical clustering** | 11/12 | 9/12 | 7/12 | 1/12 |
| --- | --- | --- | --- | --- |

Legend to S2 Table:

Rows 1 through 12 represent breast cancer paired samples of which the FFPE and FF parts were separately run on the Illumina WG-DASL V3 platform. The right four columns show the Spearman rank correlation coefficient between the expression values of the FF and FFPE materials for various *P*-values (0.01; 0.05; 0.10 and N.A. = Not Applicable). The upper row shows the numbers of probes included for the distinct levels of significance. The number of matched pairs following unsupervised hierarchical clustering is shown in the bottom row.
