# Supplementary material for: A Method to Correlate mRNA Expression Datasets Obtained from Fresh Frozen and Formalin-Fixed, Paraffin-Embedded Tissue Samples: A Matter of Thresholds
Source: PLoS One. 2015 Dec 30;10(12):e0144097. doi: 10.1371/journal.pone.0144097 (PMC4696787; doi:10.1371/journal.pone.0144097)
Supplement: S3 Table — Rows 1 through 12 represent breast cancer FFPE and FF sample pairs which were separately run on the Illumina WG-DASL V3 platform. The normalization was performed separately for the FF and FFPE samples. The right four columns show the correlation coefficient (Spearman correlation) between the expression values of FF and FFPE materials for various p-values (0.01; 0.05; 0.10 and N.A. = Not Applicable). The upper row shows the numbers of probes included for the distinct levels of significance. The number of matched pairs following unsupervised hierarchical clustering is shown in the bottom row. (DOCX) [file pone.0144097.s005.docx]

S3 Table

Title: Effect of normalization on the Spearman rank correlation between FF and FFPE samples

|  | **Number of Probes** | | | |
| --- | --- | --- | --- | --- |
| **Sample pairs** | **1,413 *P*<0.01** | **2,294 *P*<0.05** | **3,982 *P*<0.10** | **24,526 *P* N.A.** |
| Breast Ca. 1 | 0.91 | 0.86 | 0.82 | 0.57 |
| Breast Ca. 2 | 0.95 | 0.92 | 0.90 | 0.81 |
| Breast Ca. 3 | 0.97 | 0.96 | 0.95 | 0.90 |
| Breast Ca. 4 | 0.93 | 0.90 | 0.87 | 0.76 |
| Breast Ca. 5 | 0.95 | 0.93 | 0.90 | 0.82 |
| Breast Ca. 6 | 0.93 | 0.91 | 0.88 | 0.76 |
| Breast Ca. 7 | 0.95 | 0.93 | 0.91 | 0.80 |
| Breast Ca. 8 | 0.92 | 0.91 | 0.90 | 0.89 |
| Breast Ca. 9 | 0.99 | 0.99 | 0.99 | 0.98 |
| Breast Ca. 10 | 0.94 | 0.91 | 0.87 | 0.77 |
| Breast cell-line [MDA-MB-231] | 0.97 | 0.96 | 0.96 | 0.93 |
| Breast cell-line [SKBR3] | 0.96 | 0.95 | 0.94 | 0.90 |

| **Number of clustered pairs**  **following unsupervised hierarchical clustering** | 11/12 | 9/12 | 7/12 | 1/12 |
| --- | --- | --- | --- | --- |

Legend to S3 Table:

Rows 1 through 12 represent breast cancer FFPE and FF sample pairs which were separately run on the Illumina WG-DASL V3 platform. The normalization was performed separately for the FF and FFPE samples. The right four columns show the correlation coefficient (Spearman correlation) between the expression values of FF and FFPE materials for various p-values (0.01; 0.05; 0.10 and N.A. =Not Applicable). The upper row shows the numbers of probes included for the distinct levels of significance. The number of matched pairs following unsupervised hierarchical clustering is shown in the bottom row.
