# Supplementary material for: A Method to Correlate mRNA Expression Datasets Obtained from Fresh Frozen and Formalin-Fixed, Paraffin-Embedded Tissue Samples: A Matter of Thresholds
Source: PLoS One. 2015 Dec 30;10(12):e0144097. doi: 10.1371/journal.pone.0144097 (PMC4696787; doi:10.1371/journal.pone.0144097)
Supplement: S4 Table — Rows 1 through 14 represent the FFPE samples that were profiled twice on the Illumina WG-DASL V3 platform. The right four columns show the correlation coefficient (Spearman correlation) between the expression data of the duplicates for various p-values (0.01; 0.05; 0.10 and N.A. = Not Applicable). The upper row shows the numbers of probes included for the distinct levels of significance. The number of matched pairs following unsupervised hierarchical clustering is shown in the bottom row. (DOCX) [file pone.0144097.s006.docx]

S4 Table

Title: Reproducibility among repeated measurements of FFPE samples

|  | **Number of probes** | | | |
| --- | --- | --- | --- | --- |
| **duplicate samples** | **2,732 *P*<0.01** | **4,700 *P*<0.05** | **5,833 *P*<0.10** | **24,526 *P* N.A.** |
| Brain meta. lung Ca. 1 | 0.99 | 0.99 | 0.99 | 0.99 |
| Brain meta. lung Ca. 2 | 0.96 | 0.96 | 0.95 | 0.93 |
| Brain meta. lung Ca. 3 | 0.96 | 0.98 | 0.97 | 0.95 |
| Brain meta. lung Ca. 4 | 0.98 | 0.95 | 0.95 | 0.92 |
| Brain meta. lung Ca. 5 | 0.98 | 0.98 | 0.98 | 0.97 |
| Brain meta. ACUP Ca. 1 | 0.98 | 0.96 | 0.95 | 0.91 |
| Brain meta. ACUP Ca. 2 | 0.95 | 0.94 | 0.94 | 0.90 |
| Brain meta. breast Ca. | 0.99 | 0.98 | 0.99 | 0.99 |
| Brain meta. kidney Ca. | 0.96 | 0.96 | 0.96 | 0.94 |
| Brain meta. prostate Ca. | 0.88 | 0.84 | 0.83 | 0.55 |
| Brain meta. endometrium Ca. | 0.99 | 0.99 | 0.99 | 0.98 |
| Brain meta. leiomyosarcoma | 0.92 | 0.91 | 0.90 | 0.79 |
| Breast cell-line [MDA-MB-231] | 0.91 | 0.90 | 0.89 | 0.84 |
| Breast cell-line [SKBR3] | 0.97 | 0.96 | 0.95 | 0.93 |

| **Number of clustered pairs**  **following unsupervised hierarchical clustering** | 14/14 | 14/14 | 14/14 |  | 14/14 |
| --- | --- | --- | --- | --- | --- |

Legend to S4 Table:

Rows 1 through 14 represent the FFPE samples that were profiled twice on the Illumina WG-DASL V3 platform. The right four columns show the correlation coefficient (Spearman correlation) between the expression data of the duplicates for various p-values (0.01; 0.05; 0.10 and N.A. =Not Applicable). The upper row shows the numbers of probes included for the distinct levels of significance. The number of matched pairs following unsupervised hierarchical clustering is shown in the bottom row.
